# Nrf2 in Cancer, Detoxifying Enzymes and Cell Death Programs

**DOI:** 10.3390/antiox10071030

**Published:** 2021-06-25

**Authors:** Tabitha Jenkins, Jerome Gouge

**Affiliations:** Institute of Structural and Molecular Biology, Birkbeck College, University of London, London WC1E 7HX, UK; t.jenkins@bbk.ac.uk

**Keywords:** oxidative stress, Nrf2, Brf2, cancer, transcription, selenoproteins, GPx4, TrxR1, SecTRAPs

## Abstract

Reactive oxygen species (ROS) play an important role in cell proliferation and differentiation. They are also by-products of aerobic living conditions. Their inherent reactivity poses a threat for all cellular components. Cells have, therefore, evolved complex pathways to sense and maintain the redox balance. Among them, Nrf2 (Nuclear factor erythroid 2-related factor 2) plays a crucial role: it is activated under oxidative conditions and is responsible for the expression of the detoxification machinery and antiapoptotic factors. It is, however, a double edge sword: whilst it prevents tumorigenesis in healthy cells, its constitutive activation in cancer promotes tumour growth and metastasis. In addition, recent data have highlighted the importance of Nrf2 in evading programmed cell death. In this review, we will focus on the activation of the Nrf2 pathway in the cytoplasm, the molecular basis underlying Nrf2 binding to the DNA, and the dysregulation of this pathway in cancer, before discussing how Nrf2 contributes to the prevention of apoptosis and ferroptosis in cancer and how it is likely to be linked to detoxifying enzymes containing selenium.

## 1. Introduction

Reactive oxygen species (ROS) are small molecules that play important roles as mediators in cell signalling, proliferation, and differentiation [1]. They are generated during oxidative phosphorylation and metabolism, leading to ATP synthesis. Hydroxyl radical (HO^•^), hydrogen peroxide (H_2_O_2_) and superoxide anion (O_2_^•−^) are some examples of such molecules. ROS can also be generated by external factors (e.g., xenobiotic compounds and pollution) [1].

ROS are extremely reactive: they can damage lipids, nucleic acids and proteins. Lipid peroxidation is a chemical reaction occurring on polyunsaturated fatty acids that is particularly harmful to cells. Not only does it cause changes in membrane permeability and fluidity, it also creates a chain reaction that simply increases cellular ROS levels in the cells. Cells are particularly sensitive to this type of damage, and possess a specific cell death program, ferroptosis, associated with lipid peroxidation. Nucleic acids are sensitive to ROS: they can cause mutations and double strand breaks, threatening genome stability. Proteins can be oxidized, and their co-factors can be damaged or lost, all of which leads to impaired activity. This reactivity can be harnessed by cells to defend against pathogenic infection [2].

ROS are both ubiquitous by-products of our aerobic living conditions and potentially harmful molecules. Therefore, cells have evolved complex pathways to sense and maintain the redox balance or ‘redox homeostasis’. Failure to maintain this can lead to pathological conditions: ROS are implicated in several human conditions, from carcinogenesis [3] and neurodegeneration [4] to aging [5]. Redox homeostasis is maintained by a large array of enzymes that regulate detoxification. Superoxide dismutase hydrolyses O_2_^•−^, catalase and glutathione peroxidase [6] degrade H_2_O_2_, and the thioredoxin pathway reduces oxidised proteins [7]. 

**Figure 1:**
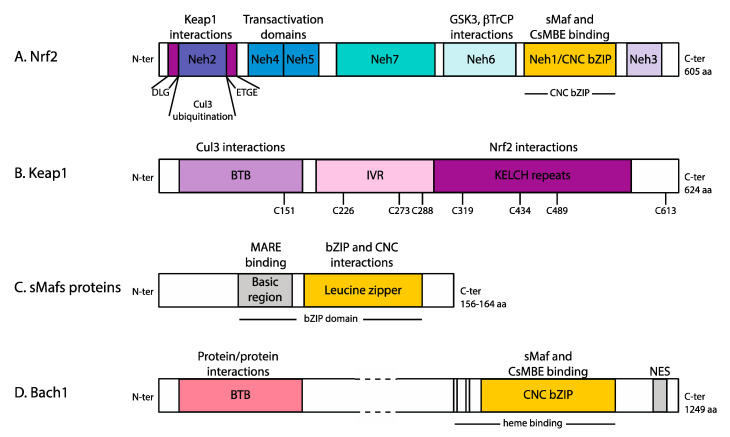
Domain representation of Nrf2, Keap1, sMaf proteins and Bach1. (**A**) Nrf2 contains a Neh2 domain that is ubiquitinated by Keap1-Cul3-Rbx1 E3 ubiquitin ligase complex. Nrf2 is recruited in the complex by the DLG and ETGE motifs flanking the Neh2. The domains Neh4 and 5 are transactivation domains, while the Neh6 domain is phosphorylated by Gsk3 (glycogen synthase kinase-3) and targeted for degradation by βTrcP. The Neh1 domain is the cap’n’collar bZIP domain, required for the interaction with sMaf proteins and DNA binding. (**B**) Keap1 contains a BTB domain required to recruit Cul3, the IVR domain that contributes to these interactions, and Kelch repeats that interact with Nrf2. Keap1 contain several reactive cysteines. (**C**) sMaf proteins are the obligate heterodimers that are required for Nrf2 to bind the DNA. They belong to the bZIP family of transcription factors. They contain a basic region that binds the DNA and leucine zipper domain that interacts with other bZIP and CNC transcription factors. (**D**) Bach1 also belongs to the cap’n’collar transcription factor family. It contains a BTB domain that allows protein/protein interactions, and a CNC domain. Additionally, it contains heme binding sites that regulate its binding on the DNA.

Several pathways are able to sense the redox balance and activate transcription of the detoxification machinery [8]. Among them, the transcription factor Nrf2 (Figure 1) is particularly important because it can be directly activated by ROS [9] and leads to the expression of the phase II detoxifying enzymes. In fact, Nrf2 (Nuclear factor erythroid 2-related factor 2) is located at the nexus between ROS signalling, endoplasmic reticulum (ER) stress response [10], metabolism [11], and autophagy [12]. It is therefore not surprising that Nrf2 target genes are involved in the antioxidant system (enzymes and co-factors), xenobiotic detoxification metabolism and excretion, DNA damage response, autophagy and antiapoptotic factors [13].

Nrf2 is regulated at the protein level by several ubiquitin ligases [9,10,11] and by post-translational modification [14,15]. In the canonical pathway and under basal conditions, Nrf2 is constitutively bound by the ubiquitin ligase Keap1-Cul3-Rbx1 [9] and targeted for proteosomal degradation. In this respect, Keap1 serves as a protein adaptor between Nrf2 and the E3 ligase. In addition, it contains several redox sensitive cysteines that modulate the conformation and activity of the complex [16]. Indeed, under oxidative conditions, the complex is inactivated and inhibits the degradation of the transcription factor. Newly synthesised Nrf2 molecules can translocate into the nucleus and express genes under its control.

Nrf2 belongs to the Cap’n’collar bZIP transcription factor family (Figure 1). In order to bind the DNA, it requires heterodimerisation with sMaf proteins. Their association in the promoter region of target genes recruits co-activators including CBP/P300, an acetyltransferase that can modify histones and transcription factors [17]. Histone acetylases are required to recruit the transcriptional machinery and relax the chromatin prior to its opening for transcription initiation. However, CBP/P300 (cAMP regulated enhancer binding protein) can also acetylate Nrf2 to increase (i) expression of the target gene [18] and (ii) the sequence specificity [19]. Interestingly, the recruitment of co-activators seems to be gene-specific: BAF190A, part of the PBAF chromatin remodeler, is specifically recruited at heme oxygenase 1 gene, but not NADPH:quinone oxidoreductase 1 [20]. Finally, Nrf2 can interact directly with Med16, a subunit of the Mediator complex, to recruit the transcriptional machinery [21].

Upon integration of different stresses, Nrf2 activation leads to the transcription of cytoprotective and antiapoptotic genes. In this regard, Nrf2 has long been considered as beneficial to protect cells against ROS damage, to help them to recover after a stress and to catabolise/excrete xenobiotic molecules. This requires tight control and regulation of the pathway. In fact, growing evidence suggest that activation of the pathway in cancer can have deleterious effects because Nrf2 acts directly or indirectly on all the hallmarks of cancer [13]. In cancer, the inherent higher ROS levels -presumably due to higher metabolism and the Warburg effect- lead to a constitutive activation of the pathway; and the same anti-apoptotic factors promote tumour growth and metastasis. The inherent higher tolerance to oxidative stress confers radio-resistance, and the xenobiotic transporters confer chemo-resistance, so that patients with cancer who have the Nrf2 pathway activated have a poor prognosis. These roles are referred to as the “dark side of Nrf2” [22]. Importantly, the mechanisms switching off Nrf2 in cancer cells, e.g., cell death programs, are also deregulated.

Some detoxifying enzymes under the control of Nrf2 are seleno-containing proteins. Selenium is a trace element that enters in the composition of some proteins, where it is present as selenomethionines (SeMet) and selenocysteines (SeCys). This class of proteins is called selenoprotein. SeCys, the 21st amino acid, is found in the three kingdoms of life but is absent in yeast, fungi and higher plants. The Human proteome contains 25 selenoproteins [23], where they are largely involved in redox regulated processes, ER stress response and calcium homeostasis. However, the function of several of them remains elusive [24]. The selenol (-SeOH) function of selenocysteine has a lower pKa (about 5.2) than cysteines (about 8.0), which makes them particularly reactive. This aspect is particularly important in respect of their activity in the oxidative stress response: two families of detoxifying enzymes, glutathione peroxidase [6] and thioredoxin reductase [25], contain a selenocysteine in their active sites. In fact, some of these detoxifying enzymes that are regulated by the oxidative stress themselves regulate the redox homeostasis and are implicated in cancer.

In this manuscript, we will review the basic mechanisms underlying Nrf2 activation in the cytoplasm as well as the recruitment of Nrf2 at its promoter. We will detail the involvement of Nrf2 in cancer development. Finally, we will discuss the latest developments in the involvement of seleno-containing detoxifying enzymes in cell death programs. 

## 2. Regulation of Nrf2 in the Cytoplasm

Nrf2 factor can be activated by different pathways. In this paragraph, we will review the different mechanisms regulating Nrf2.

### 2.1. Nrf2-Keap1-Cul3 Ubiquitin Ligase, the Canonical Pathway

The transcription factor Nrf2 is tightly regulated to ensure it only activates transcription of downstream antioxidant proteins in times of cellular oxidative stress. The major regulator of Nrf2 is the substrate adapter protein Keap1 which specifically targets Nrf2 to the E3 ubiquitin ligase complex. Keap1 (Kelch ECH-associating protein 1, Figure 1) is part of the cullin3-depedent E3 ubiquitin ligase complex which ubiquitinates Nrf2 in the cytoplasm targeting it for degradation by the 26S proteosome. We invite the reader to consult the review “structural basis of Keap1 interactions with Nrf2” for more details [26]. Under basal conditions, Nrf2 is maintained at a low cellular concentration by Keap1 activity. This allows for low levels of expression of Nrf2 regulated genes to maintain redox homeostasis [27,28]. 

**Figure 2:**
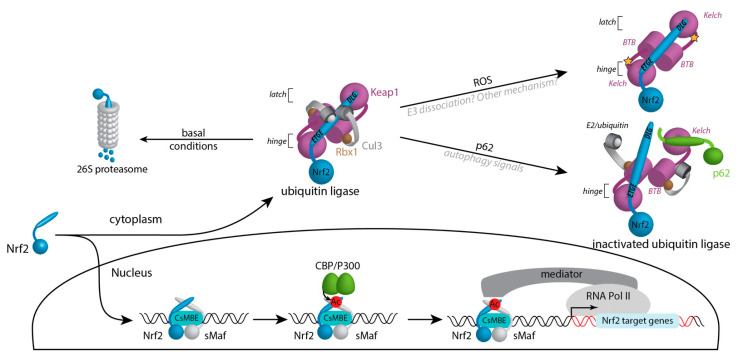
Canonical activation of the Nrf2 pathway. Nrf2 is constitutively bound by Keap1 through the DLG and ETGE motifs that serve as hinge and latch. Keap1 interacts with Cul3/Rbx1 and targets Nrf2 for proteosomal degradation. Under oxidative conditions, Keap1 can be modified at critical cysteines highlighted by a star. This inactivates the ubiquitin ligase complex by either dissociating Cul3 or a yet to be discovered mechanism. P62 can compete with the DLG motif of Nrf2 following the hinge and latch mechanism, thereby preventing ubiquitination. The newly synthesised Nrf2 molecules can translocate into the nucleus where they bind the CsMBE sequences with sMaf proteins. Nrf2 can be acetylated by CBP/P300 to increase the sequence specificity and transcriptional output. Nrf2, acetylated or not, recruits the Mediator complex and the RNA polymerase II transcriptional machinery.

Keap1 specifically interacts with two particular sequences of Nrf2 docking it for ubiquitination by the E3 ligase complex. Interactions with Keap1 occur within the Neh2 domain of Nrf2 with the Kelch domain of Keap1 (Figure 1 and Figure 2) [29]. The specific interactions between Keap1 and Nrf2 are central to the targeting of Nrf2 for ubiquitination and the regulation of Nrf2 during times of oxidative stress. Two amino acid regions within the N-terminal Neh2 domain of Nrf2 form two patches that interact with the Keap1 Kelch domain. The two motifs, ETGE and DLG, have different binding affinities for Keap1. The ETGE exhibits a slow rate of association and dissociation with a strong binding affinity compared with the DLG domain, which binds faster but with a lower binding affinity. It is hypothesised that the high affinity ETGE acts as a hinge, anchoring Nrf2 onto the Kelch, domain whereas the DLG acts as a latch; modifications to Keap1 in response to oxidative stress may prevent the latch attaching, reducing the regulatory effects that Keap1 has on Nrf2 [29,30]. This mechanism is referred to as “hinge and latch” mechanism. However, a recent report contradicts this process. Horie et al. employed NMR spectroscopy to assess the binding of labelled Neh2 domain to Keap1 [31]. The authors did not observe latch dissociation upon treatment with known electrophiles. Even if one of the compounds used was known to dissociate cullin3 (Cul3) from Keap1 [32], the others did not show this effect in previous studies [32]. The molecular mechanism underlying ubiquitination inhibition by electrophiles requires more investigation (Figure 2).

Keap1 assembles as a homodimer through their BTB domains (Figure 1 and Figure 2) into a stable and functional E3 ubiquitin ligase complex with Cul3 and Rbx1 proteins. Early reports suggested that the ubiquitin ligase complex contains two Keap1 for one Cul3/Rbx1 [33], but other works suggest a 2:2 complex [34,35]. There are a number of cullin-ring ligases (CRLs) comprised of different cullin proteins and substrate adapter proteins which are essential due to their ability to covalently modify proteins to alter their abundance and function [36]. The lysine residues targeted for poly-ubiquitination in Nrf2 are found in close proximity to the Keap1 interacting domains in the Neh2 domain [27]. Target proteins have degradation signals known as N-degrons and C-degrons situated at the N- and C-terminal of the substrate protein, respectively. These sequences contain adjoining sequence motifs with internal lysine residues primed for ubiquitination when exposed [37]. Nrf2 has an N-degron commonly found in short-lived proteins where Keap1 is able to recognise and bind Nrf2 priming the lysine residues for covalent attachment of ubiquitin. The precise mechanism of poly-ubiquitination remains to be understood at the molecular level, but what is clear is that proteasome targeting requires p97 to release Nrf2 from the ubiquitin ligase complex [38] (Figure 2). Ubiquitination is extensively studied but remains to be fully understood. For example, the roles of the possible redundant ubiquitination sites of Nrf2 remain to be clarified, as well as the stoichiometry of the complex.

Under conditions of oxidative stress, Keap1 repression of Nrf2 is prohibited. This allows Nrf2 to translocate into the nucleus and accumulate where it is free to activate transcription of antioxidant proteins to deal with the increased level of ROS [39]. The easing of repression by Keap1 relies on the ability of Keap1 to act as a redox sensor. This is achieved by the presence of several sensitive cysteine residues that are vulnerable to oxidation by electrophiles in the cell. These residues include cysteines at positions 155, 226, 272, 288, 434, 613 and particularly cysteine 151 (Figure 1 and Figure 2). They are critical in the regulation of Nrf2 and each respond to different oxidants, leading to the concept of a cysteine code [26,40]. Itaconate, a small molecule released by the mitochondria under reperfusion, has been shown in cells to react with C151 and activate the Nrf2 pathway [41]. It has been shown that a Keap1 mutant lacking 11 cysteine residues, including the ones mentioned here, is unable to respond to reactive oxidative inducers that signal for Nrf2 activation. However, these cysteine residues are not required to target Nrf2 for degradation [40]. Cysteine modifications to Keap1 during oxidative stress are thought to alter the conformation of the Cul3-Keap1-Rbx1 complex, resulting in the impaired assembly of Nrf2 into the complex and subsequent failure to ubiquitinate Nrf2. Prolonged exposure of Keap1 to oxidative stress is also thought to result in further modifications to Keap1 that expose lysine residues for its own ubiquitination [36]. Unlike ubiquitination of Nrf2, Keap1 is tagged at position lysine 63 and therefore is not targeted for proteosome degradation. Furthermore, cullin-ring ligases can be subject to auto-ubiquitination, which may be necessary to regulate levels of Nrf2 to sustain the antioxidant response over time [36]. 

Under basal cellular conditions, the Keap1 complex is the major regulator present in the cytosol turning over Nrf2 protein. Keap1 regulation of Nrf2 also occurs in the nucleus postinduction by assisting its nuclear export [42], effectively turning off Nrf2 dependent transcription.

### 2.2. Other Pathways Regulating Nrf2 Levels and Cellular Localisation

Nrf2 has also been shown to be regulated by the substrate receptor β-transducin repeat-containing protein (β-TrCP) [43]. β-TrCP is the substrate recognition subunit for the E3 ubiquitin ligase complex, Skp1-Cul1-Rbx1. This complex specifically targets phosphorylated substrates and are essential in regulating cell division and signal transduction and subsequently they are prevalent in tumorigenesis. Phosphorylation of Nrf2 in the Neh6 domain (Figure 1) catalysed by Gsk-3 creates a phosphodegron that recruits β-TrCP. β-TrCP interacts with the Skp1 E3 adaptor protein, part of the Skp1-Cul1-Rbx1 core E3 complex, which ubiquitinates Nrf2 tagging it for degradation. Two regions within the Nrf2 Neh6 region have been identified as highly conserved and are responsible for binding to β-TrCP. One site, located at the N-terminal of Neh6, called Sds1, contains a putative DSGIS non-canonical β-TrCP-binding site. The second, Sds2, is located in the C-terminal portion of Neh6 [44]. 

Nrf2 activity is also regulated by the autophagy pathway through p62 (SQSTM1). p62 competes with the DLG motif of Nrf2 [31] to binds Keap1 preventing ubiquitination of the transcription factor [12]. This pathway is regulated by post-translational modifications in which p62 is phosphorylated and ubiquitinated in order to compete with Nrf2 for Keap1 binding. SQSTM1 is activated under stress conditions acting as a scaffold protein within protein complexes to enhance signalling in DNA transcription and cell survival. It is hypothesised that p62 acts as an adaptor protein for selective autophagy, the selective removal of cellular components such as protein aggregates. p62 is activated when selected damaged components are tagged for autophagy via ubiquitination. p62 recognises ubiquitin-tags and assembles onto the complex along with other core autophagy proteins. This results in activation of early autophagosome biogenesis. During this process, p62 is phosphorylated, resulting in the destabilisation of p62 dimers. This increases the binding affinity of p62 to the ubiquitin-tagged target protein. Keap1 binds to p62 via a Keap1-interacting region (KIR), resulting in Keap1 being sequestered with the ubiquitin-tagged target protein complex, allowing the release of Nrf2 and subsequent activation. The tagged components, phosphorylated p62 and Keap1 become degraded by autophagy [12]. Furthermore, impaired autophagy can lead to increased oxidative stress, the subsequent accumulation of p62 and further upregulation of Nrf2.

Additional control of Nrf2 occurs in the nucleus to regulate its cellular localisation. These regulators include the Src subfamily A proteins, Fyn, Src, Yes and Fgr proteins that phosphorylate Nrf2 at tyrosine position 568 [45]. Phosphorylation at this site triggers Nrf2 nuclear export and subsequent cytosolic degradation to prevent prolonged permissive transcription. However, upon oxidative stress, these negative regulators are phosphorylated and exported out of the nucleus to maintain Nrf2 in the nucleus. 

## 3. Nrf2 in the Nucleus

Upon activation, e.g., repression of degradation, the newly synthesised Nrf2 molecules can translocate into the nucleus where they activate transcription of a large array of genes. In this section, we will focus on the basic principles underlying Nrf2-dependent transcription.

### 3.1. Nrf2 Target Genes

Nrf2 is a transcription factor belonging to the large basic region-leucine zipper (bZIP)-type family located in the Neh1 domain (Figure 1) [12]. Other members of this family include Jun, Fos, Bach1 and Maf proteins. This group of transcription factors is characterised by a long α-helix. The N-terminal part contains basic residues that are responsible for DNA binding and sequence specificity, the C-terminus contains several leucines responsible for homo- and heterodimerisation. In fact, heterodimerisation across bZIP transcription factors has been observed using coil-coiled arrays [46]. In addition, Nrf2 contains a conserved stretch of residues at the N-terminal of the DNA binding domain referred to as CNC (cap’n’collar) domain, which further contributes to sequence recognition. 

The complex Nrf2 dependent signalling has been extensively studied. The pathway can be activated by ROS [9] and autophagy through Keap1 phosphorylation [12] and by Gsk3/β-TrCP [11]. It is, therefore, not surprising that Nrf2 is responsible for the expression of a large array of genes implicated in the oxidative stress response and glutathione pathway, genes controlling cell proliferation and survival, metabolism, as well as proteins involved in DNA repair, drug metabolising enzymes and transporters [13]. The development of ChIP-seq techniques allowed the elucidation of the network of target genes. Using either constitutive activation (*Keap1*^−/−^) or inhibition (*Nrf2*^−/−^), Malhorta et al. identified basal and inducible target genes in mouse embryonic fibroblasts [47]. Chorley et al. used a pharmacological approach to activate the pathway and have been able to identify with high confidence new genes under the control of Nrf2 in lymphoid cells [48]. More recently, Namani et al. have been able to link Nrf2 to focal contact adhesion in cancer cells [49]. These studies clearly highlight that Nrf2 transcription is cell line and context dependent. This is important if we consider overactivation of the pathway in cancer. A particularly striking example is the ability of Nrf2 to bind enhancers of genes that are normally not activated in non-transformed cells. Okazaki et al. demonstrated that Nrf2 was able to bind the *Notch3* enhancer, recruit co-activators (as highlighted by acetylated histone marks) and activate transcription [50].

ChIP-seq experiments led to the determination of DNA sequences that Nrf2 preferentially binds. They are referred to as CsMBE (CNC-sMaf binding element) or ARE/EpRE (Antioxidant/Electrophile responsive element) [51] and are defined by (A/G)TGA(G/C)nnnGCA. These sequences are most likely derived from the MARE sequence (Maf recognition element) that are recognised by Maf proteins. 

### 3.2. Nrf2 and Its Partners Required for DNA Binding

Like other bZIP transcription factors, Nrf2 acts as a dimer. However, Nrf2 cannot bind DNA as a homodimer: it requires its obligate partner sMaf [52]. sMafs (small musculoaponeurotic fibrosarcoma) proteins are bZip transcription factors but they lack a transactivation domain (Figure 1). In this respect, they are regarded as transcriptional repressors [53]. There are three members (MafF, MafG and MafK) that can associate with Nrf2 to initiate transcription (Figure 2). Although the heterodimerisation with Nrf2 has been known for a long time, it has only recently been formally demonstrated. Using a cell line deficient for the three sMafs, Katsuoka et al. observed no induction of Nrf2 dependent genes. However, ectopic expression of a tethered dimer Nrf2/sMafG could restore transcription at Nrf2 target genes [54].

Interestingly, the consensus binding motif of sMaf (MARE: TGCTGACTCAGCA) and CsMBE are similar. The question of understanding the recognition by sMaf homodimers and sMaf/Nrf2 is important. Otsuki et al. proposed that Y502 in the Nrf2 DNA binding domain is responsible for the discrimination. Indeed, Nrf2 Y502A failed to recognise the CsMBE element while binding to the MARE sequence [51], explaining both the preferential recruitment of Nrf2/sMaf at CsMBE sequences and prevention of binding at MARE motifs.

Beyond the traditional role of Nrf2 as a transcription activator, a recent publication suggests that it can act as a repressor upon chemical activation and translocation into the nucleus. Liu et al. could demonstrate that the presence of a motif, located at the 3′end of the CsMBE in the MYLK promoter, recruits RPA1 that competes with sMaf to interact with Nrf2. Following an in silico analysis, they identified more than 420 genes containing this repressing sequence, including eEFSec that contributes to selenoprotein translation (see Section 5.4 “Nrf2 Regulation and the Selenium Connection”) [55]. This finding requires further investigation to understand the full spectrum of Nrf2 dependent transcription. 

### 3.3. Nrf2, Bach1 and the HO-1 Axis

A well-established Nrf2 axis is related to the heme oxygenase pathway. Hemes are important protein cofactors for oxygen transport and for electron transfer. In response to oxidative stress, heme is released from protein partners to induce catalysis of free radicals, acting as a pro-oxidant. Under normal cellular conditions free heme is catabolised by the heme oxygenease-1 (HO-1) enzyme. Its expression is under the control of Nrf2/sMaf, and another CNC-bZIP transcription factor Bach1. This third protein contains a BTB domain, bZIP DNA binding domain and a cytoplasmic localisation signal (Figure 1). Being a member of the CNC family, Bach1 binds the DNA with sMaf proteins although a report suggested that it can homodimerise through its BTB domain [56]. Interestingly the transactivation domain(s) of Bach1 has not been identified yet. In this respect, it is often viewed as a repressor. In fact, Bach1/sMaf bind the HO-1 promoter and represses transcription in the absence of oxidative stress or free hemes. However, Bach1 is able to sense the presence of free hemes and to bind them [57]. This affects its DNA binding capability, leading to promoter derepression [58]. Bach1 is then exported from the nucleus by Crm1 [59] where its degradation is mediated by Fbxo22 and Skp1-Cul1 [60].

Two recent publications demonstrated the role of Bach1 in lung cancer metastasis [60,61]. Two distinct laboratories demonstrated that activation of the Nrf2 pathway, either through Keap1 loss or antioxidant treatment, led to stabilisation of Bach1. Although paradoxical at first sight, because Bach1 inhibits Nrf2 transcription, this observation can be rationalised by considering the role of HO-1. Nrf2 upregulates HO-1 that in turn degrades free hemes, their absence leads to Bach1 stabilisation by inhibiting its degradation. Surprisingly, Lignitto et al. and Wiel et al. observed upregulation of pro-metastatic genes under the control of Bach1, suggesting that the previously thought repressor possesses its own transcriptional program. Wiel et al. could demonstrate that Bach1 can activate expression of Glyceraldehyde 3-phophate dehydrogenase (GAPDH) and hexokinase 2 (HK2), two proteins implicated in glucose metabolism, and is able to promote metastasis. These data illustrate that the interplay between Nrf2 and Bach1 is by far more complex than a simple activator/repressor model.

These data also open up the possibility of new cancer therapies: downregulation of Nrf2 represents a seductive approach to target cancer cells, and conversely, inhibition of Bach1 can decrease metastasis [60,61]. This is particularly relevant considering that the Nrf2 pathway is deregulated in cancer.

## 4. The Role of Nrf2 in Cancer

The development of cancer is defined by six biological abilities gained by a cell in order to break away from normal cellular behaviour and lead to tumour establishment. These are the ‘hallmarks of cancer’ as defined by Hanahan and Weinberg [62,63]. These include sustaining proliferative signalling, evading growth suppressors, resisting cell death, enabling replicative immortality, inducing angiogenesis and activating invasion and metastasis, the detachment of cells from the extracellular matrix (ECM). Furthermore, the process of metastasis has been isolated as a separate process defined as the ability for motility, invasion, colonisation and plasticity [64]. The constitutive expression of certain transcription factors enables cancer cells to gain these abilities. Just like organisms, cancer is highly complex and multi-layered which relies on a network of interactions at cellular, tissular and organismic levels [65]. The ability to break away from a primary tumour via metastasis to form multiple tumours in geographically separated tissues truly highlights its extensiveness. Cancer achieves this by interacting with multiple body systems [65]. It is often described as a systemic disease whereby the tumour-body interaction results in the primary tumour colonising host organs by expressing several factors including transcription factors. 

Transcription factors are commonly found to be up- or downregulated in the pathogenesis of cancers [66]. Cancer arises when a cell begins to proliferate abnormally as a result of genetic mutations that provide survival advantages to the cancerous cell. Mutations that are advantageous to cancer development occur predominately in two types of genes: proto-oncogenes which act as accelerators of cell cycle progression and tumour suppressor genes, which slow cellular growth. Oncogenic mutations result in a gain of function and it is estimated that about 20% of these mutations occur in transcription factor genes [67]. Tumour suppressor mutations result in a loss of function ultimately removing the barriers on cellular growth which results in uncontrolled cell division. The most widely studied tumour suppressor is the p53 transcription factor found mutated in 50% of cancers [68]. In addition, cancer cells require rewiring of the cellular metabolic pathways to provide energy and intermediates for the cancer cell to survive and thrive. Cancer cells can become vulnerable to cellular stress due to the high demand for rapid proliferation. High levels of toxic metabolic by-products, including ROS, which normal cells manage in order to maintain homeostasis, become an even more prominent issue with respect to the cancer cell viability. Therefore, cancer cells do not only upregulate proteins for growth, but also proteins required to control the detrimental by-products that come along with these advantages. It is therefore unsurprising that a prominent genetic target for cancer progression are transcription factors essential for the highly conserved signal transduction pathways. These transcription factors have become increasingly attractive targets for cancer therapy [67]. 

### 4.1. Nrf2, a Tumour Suppressor and an Oncogene

Nrf2 was originally considered as a tumour suppressor gene where a loss of function mutation aided cancer progression. Under normal cellular conditions, Nrf2 is activated within the local micro-environment in response to oxidative stress and other harmful cues. In this respect, the cytoprotective Nrf2 target genes efficiently protect cells against intracellular and external stresses: Nrf2 can be considered as a tumour suppressor [22]. 

The cytoprotective role of Nrf2 requires tight regulation and rapid inactivation to avoid deleterious side effects. Recent studies have demonstrated that Nrf2 is found hyper-activated in malignant cells with gain of function mutations resulting in survival advantages to cancer cells because the same cytoprotective genes can favour tumour growth and metastasis. Cancer cells, characterised by higher ROS levels, often show constitutive Nrf2 activation. This ultimately promotes oncogenesis by protecting tumours against oxidative stress, chemotherapeutic agents and radiotherapy [13,22,69,70]. In this respect, Nrf2 can also be considered as an oncogene. 

### 4.2. Nrf2 Pathway Is Constitutively Activated in Cancer

Cancer cells require robust ROS detoxification, it is perhaps not surprising to observe genetic mutations leading to constitutive activation of the Nrf2 pathway. This is particularly true for the regulator Keap1. The majority of mutations in Nrf2 and Keap1 identified have been found in a lung cancer background [71]. About 30% of non-small-cell lung cancer (NSCLCs) show an increase in the expression of antioxidant genes, including Nrf2, or inactivation of negative regulators, including Keap1. Nrf2 mutations are prominent occurrences in other types of lung cancer: it is mutated in 30% lung squamous cell carcinoma (LUSC) and in 25% lung adenocarcinoma (LUAC) as well as other squamous cell carcinomas. Hyper-activation of Nrf2 is associated with the worst clinical prognosis of lung cancer, highlighting it as an essential target for therapeutics in research. Furthermore, Keap1 genetic mutations have been found in 25–30% of lung cancer patients with 41% of these cases having a loss of function mutation [72]. Mutations in Keap1 that result in the stability and increased activity of Nrf2 reside within the Kelch-like repeat domain significantly reducing its binding to Nrf2 (Figure 1 and Figure 2). This reduces the ability of Keap1 to repress Nrf2 and this subsequently increases Nrf2-dependent transcription. In Nrf2, gain of function mutations are predominantly clustered in the Neh2 domain, responsible for Nrf2 regulation through interactions with the Keap1 regulator. Therefore, mutations that reduce the ability of Nrf2-Keap1 interactions result in the constitutive activation of Nrf2 and are advantageous to cancer cells [73]. It is hypothesised that Nrf2/Keap1 mutations are prominent in lung cancer because hyper-activation of Nrf2 signalling can be beneficial to lung cells constantly exposed to oxygen and chemicals from the air. Therefore, mutations that allow these cells to more readily cope with oxidative stress are not only beneficial to normal lung cells but also malignant cells [74]. 

Nrf2 is also found mutated in liver cancer with mutations found in 6.2% of hepatocellular carcinoma patients (HCC), the third biggest cause of cancer-related mortality worldwide. All mutations were found in the DLG and ETGE ‘hotspot’ motifs [75]. Little is known about the role of mutations in the Nrf2/ Keap1 pathway in the development of liver cancer due to the limited research on the development of HCC. All mutations impair Nrf2-Keap1 binding and therefore are characterised as activating mutations. However, a high frequency of Nrf2 mutations in the early steps of hepatocarcinogenesis suggests Nrf2 may be a prominent driver in the onset of HCC [76]. Furthermore, it has been recently shown that the development of HCC due to inactivation of Keap1 also relies on the activity of fructosamine-3-kinase (FN3K), which triggers de-glycation of Nrf2. When FN3K is absent, Nrf2 is extensively glycated leading to instability and an inability to interact with small Maf proteins required for transcriptional activation, ultimately providing an advantage to the cancerous cell [77]. This again highlights the great interest of Nrf2 in cancer therapy.

Mutations that target the Nrf2/Keap1 regulatory pathway are not just associated with lung and liver cancer. Keap1 mutations that prevent ubiquitination of Nrf2 have also been associated with breast, gastric, colorectal, prostate, gallbladder and ovarian cancers. Additionally, mutations in the Neh2 domain of Nrf2 have been associated with head and neck cancers, larynx, oesophagus and skin carcinomas.

As well as genetic mutations, Nrf2 hyper-activation in cancer can also arise as a result of epigenetic modifications of Keap1 [78]. Accumulation of Nrf2 can arise due to the inability of Keap1 to form stable interactions with Nrf2. One such example is the hyper-methylation of the Keap1 gene preventing its expression in lung, prostate and colorectal cancers [78,79]. Targeting the epigenetic modifications has been considered a novel strategy to increase sensitivity to anticancer drugs because of the link between the deregulation of the Nrf2 pathway with chemo-resistance.

In addition to disruption of the regulation by Keap1, Nrf2 has also been associated with carcinogenesis by interacting with proteins implicated in cancer. These proteins activate the Nrf2 pathway resulting in its constitutive activation and providing a growth advantage to malignant cells. One example is the p21 protein essential in cell-cycle arrest and oxidative stress response [80]. p21 competes with Keap1 for binding to Nrf2, stabilising and activating Nrf2. Over-expression of p21 therefore out competes Keap1 resulting in increased levels of active Nrf2 [81]. 

Predominant regulation of Nrf2 occurs at the protein level. However, Nrf2 transcription can also be increased by activated oncogenes including KRAS, BRAF and c-MYC. Therefore, Nrf2 is also described as an essential mediator of oncogenesis acting downstream of many oncogenes. The role of Nrf2 in cancer development makes it an interesting factor for therapeutic targeting. 

Cancer cells, being exposed to higher ROS levels than their healthy counterpart, leads to a constitute activation of the Nrf2 pathway. An adverse effect is the expression of anti-apoptotic factors that prevent cell death programs.

## 5. Nrf2 and the Cell Death Programs

Upon oxidative stress, Nrf2 activates the transcription of a large array of genes, including proteins involved in the thioredoxin [82] and glutathione systems [83,84]. These two systems rely on the presence of selenocysteine in the active sites of key enzymes [85]. They contribute to reduce the oxidative stress and inhibit programmed cell death. Understanding how Nrf2 contributes to evade them could constitute a strategy to treat cancer patients.

### 5.1. Detoxifying Enzymes and Cancer

Selenium is a trace element that is present in organisms as low molecular weight selenocompounds [86] and can be used as modified amino acids in proteins. Selenoproteins designate a class of proteins containing either selenomethiones (SeMet) or selenocysteines (SeCys). Selenium can be incorporated into SeMet as a function of its availability in the diet, and be used to synthetise selenomethionine-proteins. It can be catabolised in the liver to form inorganic compounds (selenate and selenide). These latter forms can be actively used by our body to incorporate selenocysteines into proteins. Considering that selenium is present at trace levels, we have evolved pathways to tune the expression of seleno-containing proteins depending on the needs and the availability of selenium. This gives rise to the so-called “selenoprotein hierarchy” [87]. 

**Figure 3:**
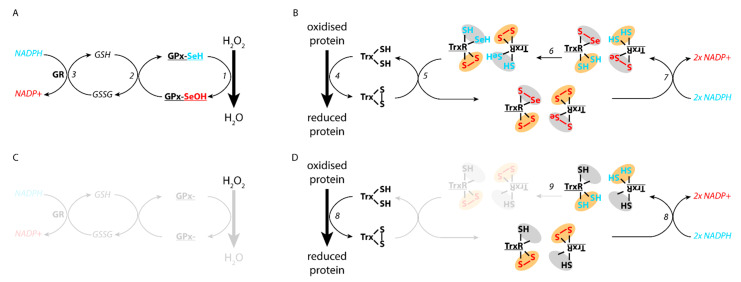
Detoxification pathways by selenoproteins glutathione peroxidase (GPx) and thiorexodin reductase 1 (TrxR1). (**A**) GPx can catabolise H_2_O_2_ upon oxidation of the selenocysteines (1). The recycling of the enzyme involves oxidation of glutathione (2), that can be reduced by glutathione reductase (GR) using NADPH as an electron donor (3). (**B**) Oxidised proteins can be reduced by oxidation of thioredoxin (Trx) (4). The recycling involves thioredoxin reductase (TrxR1) that acts as a head-to-tail homodimer. TrxR1 contains two sites; one site with 1 cysteine and 1 selenocysteine (grey in the diagram) that can reduce oxidised Trx (5). The selenothiol bond reduction is achieved by two thiols from the other monomer (orange on the diagram, 6), that are reduced by the electrons from NADPH (7). (**C**) In the absence of selenocysteine, GPx cannot reduce H_2_O_2_ because the protein lacks its catalytic residue. (**D**) In the absence of selenocysteine in TrxR1, the site containing two cysteines (in orange) can still be reduced by NADPH (8), but the electrons cannot be transferred to the selenothiol group (9). It results in a SecTRAP enzyme that depletes the levels of NADPH in cells, and increases the oxidative state. The reducing entities are highlighted in blue, the oxidised ones in red.

Selenocysteines are analogs of cysteines, with the exception that the Sulphur atom is replaced by a selenium. One of the consequences is higher nucleophilic properties, lower pKa and reduction potential that make SeCys containing proteins particularly reactive. Although some of the selenoproteins do not have known functions, most of them are involved in detoxification. The well characterised glutathione peroxidase (GPx) and thioredoxin reductase (TrxR) families are two examples of seleno-containing enzymes having antioxidant properties. We invite the readers to consult two comprehensive reviews for further details on seleno-containing proteins and their functions [23,85]. The GPx family catalyses the reduction of oxidised substrates, including H_2_O_2_ and oxidised lipids. The TrxR family is involved in the recycling of oxidised thioredoxin that has been used to reduce oxidised proteins (Figure 3). 

Possessing antioxidant functions, selenoproteins expression is intrinsically linked to the redox state. In fact, hydrogen peroxide treatment of HEK293 cells increases the expression of GPx1, GPx4 and TrxR1 as well as other selenoproteins [88]. Similarly, selenium deficiency is able to activate the Nrf2 pathway. Micro-array experiments on the intestinal cells of mice that were fed with a low Se diet demonstrate increased expression of 48 Nrf2 dependent genes, including antioxidants and phase II detoxifying enzymes, as well as increased mRNA levels of selenoproteins [89]. However this seems to be tissue specific since hepatoma cells (HepG2) do not display major changes in Nrf2 activation under selenium deficiency or supplementation [90]. We invite the reader to consult the comprehensive review from Brigelius-Flohe and Kipp [91] and Arnér [92] for more details on the crosstalk between seleno-containing detoxifying enzymes and the Nrf2 pathway.

The role of selenoproteins in cancer and metastasis has been demonstrated at different levels. The thioredoxin reductase family is generally overexpressed in cancer [93], and is correlated with poor prognosis [94]. In fact, TrxR1 is required for tumorigenesis [95]. On the contrary GPx1 expression is decreased in cancer [96], and its ectopic overexpression reduces pancreatic tumour growth both in vitro and in vivo [97]. GPx2 null mice have a higher incidence of colon tumours than wild type mice [98]. This may be due to the loss of anti-inflammatory effects of GPx2 [99]. On the contrary, the selenoprotein is overexpressed in non-inflammatory intestinal cancer [100]. Decreased expression of the secreted GPx3 has been correlated with poor prognosis in patients with prostate cancer [101]. Loss of GPx4, involved in lipid reduction, sensitises cancer cells to ferroptosis [102] (see below). The roles of detoxifying enzymes, and selenoproteins in a larger sense, in cancer are complex. In this respect, we recommend the readers interested in this aspect to consult two recent comprehensive reviews [103,104]. 

The crucial role played by detoxifying enzymes in maintaining the redox homeostasis is highlighted by cell death programs associated with their regulation.

### 5.2. Nrf2, GPx4 and Ferroptosis

Ferroptosis is a newly identified type of regulated cell death, which is independent of caspase activity [105] and is characterised by higher levels of lipid peroxidation. Lipid peroxidation is particularly harmful for cells because it threatens (i) the assembly, structure and dynamics of membranes and (ii) can further enhance the ROS levels through Fenton reaction [106]. Cells rely on a complex machinery to detect lipids peroxidation and their reduction. The selenoprotein glutathione perodixase 4 (GPx4) is particularly important in that aspect: the protein catalyses the reduction of oxidised lipids using glutathione as an electron donor. The oxidised glutathione is then recycled by a glutathione reductase using NADPH (Figure 3). If either the glutathione biogenesis pathway or GPx4 is compromised, ferroptosis can be triggered [107]. Considering that GPx4 is a bona fide target gene of Nrf2, either directly or indirectly, several links between ferroptosis and the Nrf2 pathway have been proposed. We would like to direct the reader to a recent review for more details [108]. 

Of particular interest, Takahashi et al. [109] recently used CRISPR-Cas9 screens to identify genes required by Nrf2 hyperactivation to sustain cell proliferation in 3D spheroids. This culture technique recapitulates the gradients naturally occurring in tumour (nutrients, oxygen, metabolism [110]). Using lung cancer cell lines (A549 and H1347), they demonstrated that Nrf2 inhibition by shRNA reduces cellular proliferation more significantly in 3D than in conventional cell culture. Importantly, Nrf2 knockdown decreases cell proliferation at the early stage, and clearance of the inner core at the later stages. Conversely the authors also demonstrated that Nrf2 activation increases cell survival of the inner core through ferroptosis prevention. In fact, knocking down both Nrf2 and GPx4 was more lethal in both A549 and H1347 spheroids than 2D.

It is now clear that tumours require inhibition of ferroptosis to sustain proliferation [111]. Inhibition of both the Nrf2 pathway and GPx4 activity (or glutathione pathway) is an attractive strategy to specifically target cancer cells.

### 5.3. Nrf2, TrxR1 and Apoptosis

Nrf2 is responsible for the expression of thioredoxin reductase 1 (TrxR1). TrxR1 is part of the thioredoxin system. Thioredoxins are small proteins that catalyse the reduction of proteins by cysteine thiol-disulfide exchange. TrxR1 activity is necessary to recycle thioredoxin (Trx) [25]. The electrons from NADPH are transferred to an oxidised Trx in several steps: first through FAD then to a disulfide bond in the N-terminal domain of molecule A. They are then transferred to a second site located at the C-terminal part of molecule B that contains a selenylsulfide (Se-S) covalent bond. The selenothiol can then reduce oxidised Trx to recycle the molecule (Figure 3). The presence of a selenothiol group is of utmost importance for the correct activity of TrxR1.

As recently noted by Arner [112], the role of TrxR1 is complex in cancer, but compounds targeting the selenocysteine in TrxR1 have been developed and demonstrated reduction in cancer cell viability both in vitro and in mouse models [113,114].

Compounds modifying the selenocysteine in TrxR1 are thought to transform the detoxifying enzyme into a pro-oxidant protein. The term used by Anestal et al. is SecTRAPs for selenium compromised thioredoxin reductase-deprived apoptotic proteins. Under conditions where the selenocysteines are compromised, either by nucleophile reaction or truncation, SecTRAPs can still utilise NADPH to reduce FAD but the electron transfer to Trx is interrupted. It results in production of ROS in a feed forward mechanism that ultimately leads to apoptosis [115,116].

It appears that the presence of selenocysteines in both GPx4 and TrxR1 is necessary to sustain detoxification [115,116,117] and prevent triggering of cell death programs.

### 5.4. Nrf2 Regulation and the Selenium Connection

Nrf2 couples expression of the phase II detoxifying enzymes, including GPx4 and TrxR1, with expression of anti-apoptotic factors [118]. It seems paradoxical to observe cell death programs triggered even under Nrf2 activation. It is therefore important to understand how cell ferroptosis and apoptosis are triggered in a healthy context. This knowledge could potentially be applied to target cancer cells that often show Nrf2 activation and inhibition of ROS-related cell death programs. In the next paragraphs, we will discuss the possible mechanisms underlying the control of programmed cell death in heathy cells and how they are deregulated in cancer. 

The selenocysteines are encoded by the UGA stop codon. Cells rely on a specific machinery to properly insert these residues during translation. A specific stem-loop on the mRNA, called SECIS element, is recognised by SBP2 that in turn recruits eEFsec (selenocysteine-specific elongation factor). eEFsec also binds the SeCys tRNA and ensures efficient incorporation of the amino acid into the selenoproteins [85]. 

Importantly, depletion of SBP2 or eEFSec leads to lower levels of GPx4 [119]. Furthermore, Papp et al. demonstrated that SBP2 knockdown increases ROS levels and apoptosis [120]. This highlights that defects in the selenocysteine pathway have tremendous effects on the synthesis of detoxifying enzymes, and ultimately the oxidative stress response and cell survival.

**Figure 4:**
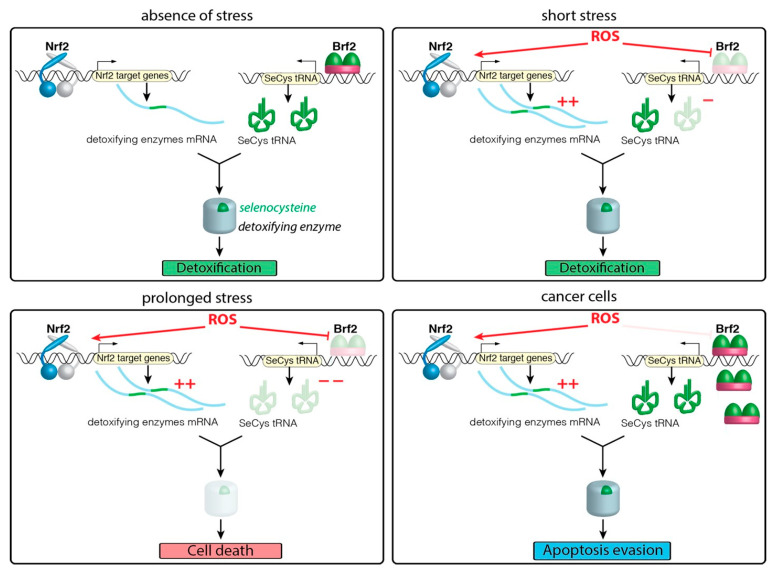
In the absence of stress, basal levels of Nrf2 stimulate the expression of the detoxifying enzymes, the translation of which requires the selenocysteine tRNAs (SeCys tRNA) under the control of Brf2. In the presence of a short stress, the Nrf2 pathway is activated by inhibition of Keap1 dependent degradation. Under the same conditions, Brf2 doesn’t bind the DNA and prevent the translation of the SeCys tRNA. The cells have to use the pre-existing pool of tRNA to translate the detoxifying enzymes. Under prolonged oxidative conditions, the SeCys tRNA become the limiting factor of the detoxifying enzymes translation. This ultimately triggers the cell death programs. Cancer cells overexpress Brf2 to bypass the limiting step and maintain translation of the detoxifying enzymes and evade cell death.

A recent report suggests that the SeCys tRNA transcription is under a redox control. The authors provided a model explaining the crosstalk between Nrf2 activation and redox control of the detoxifying enzyme translation [121]. Transcription of the SeCys tRNA is achieved by the RNA polymerase III, which is responsible for the expression of all the short and untranslated RNA, including the entire pool of tRNA. More specifically, the SeCys tRNAs are transcribed at the type 3 promoters in metazoans [122]. These promoters are markedly different from the type 1 and 2 in their architecture and requirement of specific transcription factors [123]. Recruitment of the RNA Polymerase III relies on TFIIIB, a core transcription factor composed of three subunits: (i) TBP, (ii) Bdp1 [124], and (iii) Brf2 (B-related factor 2). Recent studies have highlighted a direct link between Brf2 over-expression and many types of cancer, including lung [125,126] and breast cancer [127,128]. The crystal structure of Brf2 in complex with TBP and the DNA revealed the presence of a conserved cysteine interacting with DNA [121]. Gouge et al. demonstrated that, under oxidative conditions, the cysteine can be oxidised and abrogates Brf2 binding on the DNA. This negatively regulates the RNA polymerase III recruitment specifically at the type 3 promoters, thereby modulating the transcriptional output, including the SeCys tRNA. Gouge et al. could demonstrate that the lack of SeCys tRNA availability combined with its relative short half-life [129] contributes to the regulation the selenoproteins levels, including GPx4. In fact, it was proposed that under prolonged oxidative conditions, the SeCys tRNAs become the limiting factor in the selenoprotein translation: the authors observed appearance of truncated forms of selenoproteins upon sustained oxidative treatment. As expected, the truncated detoxifying enzymes triggered apoptosis in healthy cells. Conversely, Brf2 knockdown in A459 cancer cells, in which the Nrf2 pathway is overactivated and Brf2 overexpressed, leads to cell death. They proposed that Brf2 acts as a master switch in the oxidative stress response by regulating in a redox dependent manner the levels of SeCys tRNA. This safety mechanism is by-passed in cancer: by overexpressing Brf2, cancer cells alleviate this regulation and prevent apoptosis. Importantly, this phenomenon occurs even in the presence of activated Nrf2 [130] (Figure 4). 

In light of the growing body of evidence linking GPx4 deficiency and ferroptosis, the role of Brf2 in this particular pathway needs to be clarified, especially in 3D spheroid cultures. In a broader context, triggering one cell death pathway over the other (apoptosis versus ferroptosis) remains an open question but it is likely to involve a feedforward mechanism. Under prolonged oxidative levels, appearance of truncated detoxifying enzymes could lead to further increased ROS levels. Under high oxidative conditions, Nrf2 can express the transcription factor Kruppel-like factor 9 (Klf9) [131]. In turn, accumulation of Klf9 in the nucleus suppresses the expression of TrxR2, which further increases ROS levels. Zucker et al. demonstrated that this is sufficient to trigger apoptosis [131]. Considering that the cell programs can be triggered by different compromised detoxifying enzymes (GPx4 and SecTRAPs), it is possible that the preferential activation of one pathway over the other might be linked to their expression levels in different tissues and cell types, as well as their position in the “selenoprotein hierarchy”. In addition, a possible crosstalk between SecTRAPs and ferroptosis is also possible, as high doses of auranofin, an inhibitor of TrxR family, can induce ferroptosis in mice liver [132]. Further research on the conditions leading to ferroptosis or apoptosis is required to explain their preferential activation.

## 6. Conclusions

Nrf2 possesses undoubtedly beneficial roles in cancer prevention by activating the ROS detoxification machinery, antiapoptotic genes, xenobiotic metabolising enzymes, and transporters. This pathway requires tight control because, upon deregulation, it promotes metastasis and tumour growth, ultimately threatening cancer patient survival. Deregulation of Nrf2 in cancer can arise from several causes: from inherent higher ROS levels, to point mutations and promoter silencing. This leads to apoptosis and ferroptosis prevention. In this respect, efforts to develop small molecules modulating the Nrf2 pathway have been ongoing for the last few years, and the focus has recently shifted from activating to inhibiting the pathway [133]. However, one would expect side effects associated with modulators of the oxidative stress response. A possible strategy would be to re-activate the cell death programs or use a combination of drugs targeting both the Nrf2 pathway and the detoxification machinery. Obtaining high resolution structures along the Nrf2 pathway, deepening understanding of Nrf2 crosstalk with the transcriptional machinery, and characterisation of the cell death programs would, hopefully, allow more targeted cancer therapies. Detoxifying enzymes containing a catalytic selenocysteine are regulated by and regulate ROS levels. Taking into account that they possess the ability to trigger cell death programs when they are compromised, cancer cells need to by-pass this regulation by overexpressing them and/or sustaining SeCys tRNA levels. Future research might also concentrate on further characterising the functions of seleno-containing proteins and their intricate relationship with the Nrf2 pathway. This would allow for the development of therapies for the benefit of cancer patients.

## References

[1] Ray P.D., Huang B.-W., Tsuji Y. (2012). Reactive oxygen species (ROS) homeostasis and redox regulation in cellular signaling. Cell. Signal..

[2] Paiva C.N., Bozza M. (2014). Are Reactive Oxygen Species Always Detrimental to Pathogens?. Antioxid. Redox Signal..

[3] Trachootham D., Alexandre J., Huang P. (2009). Targeting cancer cells by ROS-mediated mechanisms: A radical therapeutic approach?. Nat. Rev. Drug Discov..

[4] Andersen J.K. (2004). Oxidative stress in neurodegeneration: Cause or consequence?. Nat. Med..

[5] Haigis M.C., Yankner B.A. (2010). The Aging Stress Response. Mol. Cell.

[6] Brigelius-Flohé R., Maiorino M. (2013). Glutathione peroxidases. Biochim. Biophys. Acta.

[7] Holmgren A., Lu J. (2010). Thioredoxin and thioredoxin reductase: Current research with special reference to human disease. Biochem. Biophys. Res. Commun..

[8] Brigelius-Flohé R., Flohé L. (2011). Basic Principles and Emerging Concepts in the Redox Control of Transcription Factors. Antioxid. Redox Signal..

[9] Itoh K., Wakabayashi N., Katoh Y., Ishii T., O’Connor T., Yamamoto M. (2003). Keap1 regulates both cytoplasmic-nuclear shuttling and degradation of Nrf2 in response to electrophiles. Genes Cells.

[10] Wu T., Zhao F., Gao B., Tan C., Yagishita N., Nakajima T., Wong P.K., Chapman E., Fang D., Zhang D.D. (2014). Hrd1 suppresses Nrf2-mediated cellular protection during liver cirrhosis. Genes Dev..

[11] Rada P., Rojo A.I., Chowdhry S., McMahon M., Hayes J., Cuadrado A. (2011). SCF/ -TrCP Promotes Glycogen Synthase Kinase 3-Dependent Degradation of the Nrf2 Transcription Factor in a Keap1-Independent Manner. Mol. Cell. Biol..

[12] Katsuragi Y., Ichimura Y., Komatsu M. (2016). Regulation of the Keap1–Nrf2 pathway by p62/SQSTM1. Curr. Opin. Toxicol..

[13] De La Vega M.R., Chapman E., Zhang D.D. (2018). NRF2 and the Hallmarks of Cancer. Cancer Cell.

[14] Liu T., Lv Y.-F., Zhao J.-L., You Q.-D., Jiang Z.-Y. (2021). Regulation of Nrf2 by phosphorylation: Consequences for biological function and therapeutic implications. Free. Radic. Biol. Med..

[15] Walters T.S., McIntosh D.J., Ingram S.M., Tillery L., Motley E.D., Arinze I.J., Misra S. (2021). SUMO-Modification of Human Nrf2 at K110 and K533 Regulates Its Nucleocytoplasmic Localization, Stability and Transcriptional Activity. Cell. Physiol. Biochem..

[16] Baird L., Llères D., Swift S., Dinkova-Kostova A.T. (2013). Regulatory flexibility in the Nrf2-mediated stress response is conferred by conformational cycling of the Keap1-Nrf2 protein complex. Proc. Natl. Acad. Sci. USA.

[17] Dancy B.M., Cole P.A. (2015). Protein Lysine Acetylation by p300/CBP. Chem. Rev..

[18] Kawai Y., Garduño L., Theodore M., Yang J., Arinze I.J. (2011). Acetylation-Deacetylation of the Transcription Factor Nrf2 (Nuclear Factor Erythroid 2-related Factor 2) Regulates Its Transcriptional Activity and Nucleocytoplasmic Localization. J. Biol. Chem..

[19] Sun Z., Chin Y.E., Zhang D.D. (2009). Acetylation of Nrf2 by p300/CBP Augments Promoter-Specific DNA Binding of Nrf2 during the Antioxidant Response. Mol. Cell. Biol..

[20] Zhang J., Ohta T., Maruyama A., Hosoya T., Nishikawa K., Maher J.M., Shibahara S., Itoh K., Yamamoto M. (2006). BRG1 Interacts with Nrf2 To Selectively Mediate HO-1 Induction in Response to Oxidative Stress. Mol. Cell. Biol..

[21] Sekine H., Okazaki K., Ota N., Shima H., Katoh Y., Suzuki N., Igarashi K., Ito M., Motohashi H., Yamamoto M. (2016). The Mediator Subunit MED16 Transduces NRF2-Activating Signals into Antioxidant Gene Expression. Mol. Cell. Biol..

[22] Menegon S., Columbano A., Giordano S. (2016). The Dual Roles of NRF2 in Cancer. Trends Mol. Med..

[23] Johansson L., Gafvelin G., Arnér E.S. (2005). Selenocysteine in proteins—Properties and biotechnological use. Biochim. Biophys. Acta (BBA) Gen. Subj..

[24] Kasaikina M.V., Hatfield L.L., Gladyshev V.N. (2012). Understanding selenoprotein function and regulation through the use of rodent models. Biochim. Biophys. Acta (BBA) Bioenerg..

[25] Lu J., Holmgren A. (2014). The thioredoxin antioxidant system. Free Radic. Biol. Med..

[26] Canning P., Sorrell F.J., Bullock A.N. (2015). Structural basis of Keap1 interactions with Nrf2. Free Radic. Biol. Med..

[27] Zhang D.D., Lo S.-C., Cross J.V., Templeton D.J., Hannink M. (2004). Keap1 Is a Redox-Regulated Substrate Adaptor Protein for a Cul3-Dependent Ubiquitin Ligase Complex. Mol. Cell. Biol..

[28] Cullinan S.B., Gordan J.D., Jin J., Harper J.W., Diehl J.A. (2004). The Keap1-BTB Protein Is an Adaptor That Bridges Nrf2 to a Cul3-Based E3 Ligase: Oxidative Stress Sensing by a Cul3-Keap1 Ligase. Mol. Cell Biol..

[29] Tong K.I., Padmanabhan B., Kobayashi A., Shang C., Hirotsu Y., Yokoyama S., Yamamoto M. (2007). Different Electrostatic Potentials Define ETGE and DLG Motifs as Hinge and Latch in Oxidative Stress Response. Mol. Cell. Biol..

[30] Tong K.I., Kobayashi A., Katsuoka F., Yamamoto M. (2006). Two-site substrate recognition model for the Keap1-Nrf2 system: A hinge and latch mechanism. Biol. Chem..

[31] Horie Y., Suzuki T., Inoue J., Iso T., Wells G., Moore T.W., Mizushima T., Dinkova-Kostova A.T., Kasai T., Kamei T. (2021). Molecular basis for the disruption of Keap1–Nrf2 interaction via Hinge & Latch mechanism. Commun. Biol..

[32] Iso T., Suzuki T., Baird L., Yamamoto M. (2016). Absolute Amounts and Status of the Nrf2-Keap1-Cul3 Complex within Cells. Mol. Cell. Biol..

[33] Small E., Eggler A., Mesecar A.D. (2010). Development of an efficient E. coli expression and purification system for a catalytically active, human Cullin3–RINGBox1 protein complex and elucidation of its quaternary structure with Keap1. Biochem. Biophys. Res. Commun..

[34] Zhuang M., Calabrese M.F., Liu J., Waddell M.B., Nourse A., Hammel M., Miller D.J., Walden H., Duda D.M., Seyedin S.N. (2009). Structures of SPOP-Substrate Complexes: Insights into Molecular Architectures of BTB-Cul3 Ubiquitin Ligases. Mol. Cell.

[35] Choo Y.Y., Hagen T. (2012). Mechanism of Cullin3 E3 Ubiquitin Ligase Dimerization. PLoS ONE.

[36] Villeneuve N.F., Lau A., Zhang D.D. (2010). Regulation of the Nrf2–Keap1 Antioxidant Response by the Ubiquitin Proteasome System: An Insight into Cullin-Ring Ubiquitin Ligases. Antioxid. Redox Signal..

[37] Varshavsky A. (2019). N-degron and C-degron pathways of protein degradation. Proc. Natl. Acad. Sci. USA.

[38] Tao S., Liu P., Luo G., de la Vega M.R., Chen H., Wu T., Tillotson J., Chapman E., Zhang D.D. (2017). p97 Negatively Regulates NRF2 by Extracting Ubiquitylated NRF2 from the KEAP1-CUL3 E3 Complex. Mol. Cell. Biol..

[39] Lee D.-F., Kuo H.-P., Liu M., Chou C.-K., Xia W., Du Y., Shen J., Chen C.-T., Huo L., Hsu M.-C. (2009). KEAP1 E3 Ligase-Mediated Downregulation of NF-κB Signaling by Targeting IKKβ. Mol. Cell.

[40] Suzuki T., Muramatsu A., Saito R., Iso T., Shibata T., Kuwata K., Kawaguchi S.-I., Iwawaki T., Adachi S., Suda H. (2019). Molecular Mechanism of Cellular Oxidative Stress Sensing by Keap1. Cell Rep..

[41] Mills E.L., Ryan D.G., Prag H.A., Dikovskaya D., Menon D., Zaslona Z., Jedrychowski M.P., Costa A.S.H., Higgins M., Hams E. (2018). Itaconate Is an Anti-Inflammatory Metabolite That Activates Nrf2 via Alkylation of KEAP1. Nature.

[42] Sun Z., Zhang S., Chan J.Y., Zhang D.D. (2007). Keap1 Controls Postinduction Repression of the Nrf2-Mediated Antioxidant Response by Escorting Nuclear Export of Nrf2. Mol. Cell. Biol..

[43] Chowdhry S., Zhang Y., McMahon M., Sutherland C., Cuadrado A., Hayes J.D. (2013). Nrf2 is controlled by two distinct β-TrCP recognition motifs in its Neh6 domain, one of which can be modulated by GSK-3 activity. Oncogene.

[44] Fuchs S.Y., Spiegelman V.S., Kumar K.G.S. (2004). The many faces of β-TrCP E3 ubiquitin ligases: Reflections in the magic mirror of cancer. Oncogene.

[45] Niture S., Khatri R., Jaiswal A.K. (2014). Regulation of Nrf2—An update. Free Radic. Biol. Med..

[46] Newman J.R.S. (2003). Comprehensive Identification of Human bZIP Interactions with Coiled-Coil Arrays. Science.

[47] Malhotra D., Portales-Casamar E., Singh A., Srivastava S., Arenillas D., Happel C., Shyr C., Wakabayashi N., Kensler T.W., Wasserman W.W. (2010). Global mapping of binding sites for Nrf2 identifies novel targets in cell survival response through ChIP-Seq profiling and network analysis. Nucleic Acids Res..

[48] Chorley B.N., Campbell M.R., Wang X., Karaca M., Sambandan D., Bangura F., Xue P., Pi J., Kleeberger S., Bell D.A. (2012). Identification of novel NRF2-regulated genes by ChIP-Seq: Influence on retinoid X receptor alpha. Nucleic Acids Res..

[49] Namani A., Liu K., Wang S., Zhou X., Liao Y., Wang H., Wang X.J., Tang X. (2019). Genome-wide global identification of NRF2 binding sites in A549 non-small cell lung cancer cells by ChIP-Seq reveals NRF2 regulation of genes involved in focal adhesion pathways. Aging.

[50] Okazaki K., Anzawa H., Liu Z., Ota N., Kitamura H., Onodera Y., Alam M., Matsumaru D., Suzuki T., Katsuoka F. (2020). Enhancer remodeling promotes tumor-initiating activity in NRF2-activated non-small cell lung cancers. Nat. Commun..

[51] Otsuki A., Suzuki M., Katsuoka F., Tsuchida K., Suda H., Morita M., Shimizu R., Yamamoto M. (2016). Unique cistrome defined as CsMBE is strictly required for Nrf2-sMaf heterodimer function in cytoprotection. Free Radic. Biol. Med..

[52] Itoh K., Chiba T., Takahashi S., Ishii T., Igarashi K., Katoh Y., Oyake T., Hayashi N., Satoh K., Hatayama I. (1997). An Nrf2/Small Maf Heterodimer Mediates the Induction of Phase II Detoxifying Enzyme Genes through Antioxidant Response Elements. Biochem. Biophys. Res. Commun..

[53] Katsuoka F., Yamamoto M. (2016). Small Maf proteins (MafF, MafG, MafK): History, structure and function. Gene.

[54] Katsuoka F., Otsuki A., Takahashi M., Ito S., Yamamoto M. (2019). Direct and Specific Functional Evaluation of the Nrf2 and MafG Heterodimer by Introducing a Tethered Dimer into Small Maf-Deficient Cells. Mol. Cell. Biol..

[55] Liu P., de la Vega M.R., Sammani S., Mascarenhas J.B., Kerins M., Dodson M., Sun X., Wang T., Ooi A., Garcia J.G.N. (2018). RPA1 binding to NRF2 switches ARE-dependent transcriptional activation to ARE-NRE–dependent repression. Proc. Natl. Acad. Sci. USA.

[56] Ito N., Igarashi K., Murayama K., Watanabe-Matsui M. (2009). Crystal structure of the Bach1 BTB domain and its regulation of homodimerization. Genes Cells.

[57] Ogawa K., Sun J., Taketani S., Nakajima O., Nishitani C., Sassa S., Hayashi N., Yamamoto M., Shibahara S., Fujita H. (2001). Heme mediates derepression of Maf recognition element through direct binding to transcription repressor Bach1. EMBO J..

[58] Sun J., Hoshino H., Takaku K., Nakajima O., Muto A., Suzuki H., Tashiro S., Takahashi S., Shibahara S., Alam J. (2002). Hemoprotein Bach1 regulates enhancer availability of heme oxygenase-1 gene. EMBO J..

[59] Cai Y., Li B., Peng D., Wang X., Li P., Huang M., Xing H., Chen J. (2021). Crm1-Dependent Nuclear Export of Bach1 is Involved in the Protective Effect of Hyperoside on Oxidative Damage in Hepatocytes and CCl4-induced Acute Liver Injury. J. Inflamm. Res..

[60] Lignitto L., Leboeuf S.E., Homer H., Jiang S., Askenazi M., Karakousi T.R., Pass H., Bhutkar A.J., Tsirigos A., Ueberheide B. (2019). Nrf2 Activation Promotes Lung Cancer Metastasis by Inhibiting the Degradation of Bach1. Cell.

[61] Wiel C., Le Gal K., Ibrahim M., Jahangir C.A., Kashif M., Yao H., Ziegler D., Xu X., Ghosh T., Mondal T. (2019). BACH1 Stabilization by Antioxidants Stimulates Lung Cancer Metastasis. Cell.

[62] Hanahan D., Weinberg R.A. (2000). The Hallmarks of Cancer. Cell.

[63] Hanahan D., Weinberg R.A. (2011). Hallmarks of Cancer: The Next Generation. Cell.

[64] Nguyen D.X., Massague J. (2007). Genetic determinants of cancer metastasis. Nat. Rev. Genet..

[65] Paul D. (2020). The systemic hallmarks of cancer. J. Cancer Metastasis Treat..

[66] Bhagwat A.S., Vakoc C.R. (2015). Targeting Transcription Factors in Cancer. Trends Cancer.

[67] Nebert D.W. (2002). Transcription factors and cancer: An overview. Toxicology.

[68] Vishnoi K., Viswakarma N., Rana A., Rana B. (2020). Transcription Factors in Cancer Development and Therapy. Cancers.

[69] DeNicola G.M., Karreth F.A., Humpton T., Gopinathan A., Wei C., Frese K., Mangal D., Yu K.H., Yeo C.J., Calhoun E.S. (2011). Oncogene-induced Nrf2 transcription promotes ROS detoxification and tumorigenesis. Nat. Cell Biol..

[70] Gañán-Gómez I., Wei Y., Yang H., Boyano-Adánez M.C., García-Manero G. (2013). Oncogenic functions of the transcription factor Nrf2. Free Radic. Biol. Med..

[71] Ohta T., Iijima K., Miyamoto M., Nakahara I., Tanaka H., Ohtsuji M., Suzuki T., Kobayashi A., Yokota J., Sakiyama T. (2008). Loss of Keap1 Function Activates Nrf2 and Provides Advantages for Lung Cancer Cell Growth. Cancer Res..

[72] Singh A., Misra V., Thimmulappa R.K., Lee H., Ames S., Hoque M.O., Herman J.G., Baylin S.B., Sidransky D., Gabrielson E. (2006). Dysfunctional KEAP1–NRF2 Interaction in Non-Small-Cell Lung Cancer. PLoS Med..

[73] Shibata T., Ohta T., Tong K.I., Kokubu A., Odogawa R., Tsuta K., Asamura H., Yamamoto M., Hirohashi S. (2008). Cancer related mutations in NRF2 impair its recognition by Keap1-Cul3 E3 ligase and promote malignancy. Proc. Natl. Acad. Sci. USA.

[74] Siegel D., A Franklin W., Ross D. (1998). Immunohistochemical detection of NAD(P)H:quinone oxidoreductase in human lung and lung tumors. Clin. Cancer Res..

[75] Guichard C., Amaddeo G., Imbeaud S., Ladeiro Y., Pelletier L., Ben Maad I., Calderaro J., Bioulac-Sage P., Letexier M., Degos F. (2012). Integrated analysis of somatic mutations and focal copy-number changes identifies key genes and pathways in hepatocellular carcinoma. Nat. Genet..

[76] Kowalik M.A., Guzzo G., Morandi A., Perra A., Menegon S., Masgras I., Trevisan E., Angioni M.M., Fornari F., Quagliata L. (2016). Metabolic reprogramming identifies the most aggressive lesions at early phases of hepatic carcinogenesis. Oncotarget.

[77] Sanghvi V.R., Leibold J., Mina M., Mohan P., Berishaj M., Li Z., Miele M.M., Lailler N., Zhao C., de Stanchina E. (2019). The Oncogenic Action of NRF2 Depends on De-glycation by Fructosamine-3-Kinase. Cell.

[78] Wang R., An J., Ji F., Jiao H., Sun H., Zhou D. (2008). Hypermethylation of the Keap1 gene in human lung cancer cell lines and lung cancer tissues. Biochem. Biophys. Res. Commun..

[79] Muscarella L.A., Parrella P., D’Alessandro V., la Torre A., Barbano R., Fontana A., Tancredi A., Guarnieri V., Balsamo T., Coco M. (2011). Frequent epigenetics inactivation of KEAP1 gene in non-small cell lung cancer. Epigenetics.

[80] O’Reilly M.A. (2005). Redox Activation of p21Cip1/WAF1/Sdi1: A Multifunctional Regulator of Cell Survival and Death. Antioxid. Redox Signal..

[81] Chen W., Sun Z., Wang X.-J., Jiang T., Huang Z., Fang D., Zhang D.D. (2009). Direct Interaction between Nrf2 and p21Cip1/WAF1 Upregulates the Nrf2-Mediated Antioxidant Response. Mol. Cell.

[82] Sakurai A., Nishimoto M., Himeno S., Imura N., Tsujimoto M., Kunimoto M., Hara S. (2005). Transcriptional regulation of thioredoxin reductase 1 expression by cadmium in vascular endothelial cells: Role of NF-E2-related factor-2. J. Cell. Physiol..

[83] Thimmulappa R.K., Mai K.H., Srisuma S., Kensler T.W., Yamamoto M., Biswal S. (2002). Identification of Nrf2-regulated genes induced by the chemopreventive agent sulforaphane by oligonucleotide microarray. Cancer Res..

[84] Kwak M.-K., Wakabayashi N., Itoh K., Motohashi H., Yamamoto M., Kensler T.W. (2003). Modulation of Gene Expression by Cancer Chemopreventive Dithiolethiones through the Keap1-Nrf2 Pathway. J. Biol. Chem..

[85] Papp L.V., Lu J., Holmgren A., Khanna K.K. (2007). From Selenium to Selenoproteins: Synthesis, Identity, and Their Role in Human Health. Antioxid. Redox Signal..

[86] Flouda K., Dersch J.M., Gabel-Jensen C., Stürup S., Misra S., Björnstedt M., Gammelgaard B. (2016). Quantification of low molecular weight selenium metabolites in human plasma after treatment with selenite in pharmacological doses by LC-ICP-MS. Anal. Bioanal. Chem..

[87] Schomburg L., Schweizer U. (2009). Hierarchical regulation of selenoprotein expression and sex-specific effects of selenium. Biochim. Biophys. Acta (BBA) Gen. Subj..

[88] Touat-Hamici Z., Legrain Y., Bulteau A.-L., Chavatte L. (2014). Selective Up-regulation of Human Selenoproteins in Response to Oxidative Stress. J. Biol. Chem..

[89] Muller M., Banning A., Brigelius-Flohé R., Kipp A. (2010). Nrf2 target genes are induced under marginal selenium-deficiency. Genes Nutr..

[90] Tauber S., Sieckmann M., Erler K., Stahl W., Klotz L.-O., Steinbrenner H. (2021). Activation of Nrf2 by Electrophiles Is Largely Independent of the Selenium Status of HepG2 Cells. Antioxidants.

[91] Brigelius-Flohé R., Kipp A.P., Cadenas E., Packer L. (2013). Chapter Four—Selenium in the Redox Regulation of the Nrf2 and the Wnt Pathway. Methods in Enzymology.

[92] Arnér E.S.J., Schmidt H.H.H.W., Ghezzi P., Cuadrado A. (2021). Effects of Mammalian Thioredoxin Reductase Inhibitors. Reactive Oxygen Species: Network Pharmacology and Therapeutic Applications.

[93] Selenius M., Rundlöf A.-K., Olm E., Fernandes A.P., Björnstedt M. (2010). Selenium and the Selenoprotein Thioredoxin Reductase in the Prevention, Treatment and Diagnostics of Cancer. Antioxid. Redox Signal..

[94] Bhatia M., McGrath K.L., Di Trapani G., Charoentong P., Shah F., King M.M., Clarke F.M., Tonissen K.F. (2016). The thioredoxin system in breast cancer cell invasion and migration. Redox Biol..

[95] Yoo M.-H., Xu X.-M., Carlson B.A., Gladyshev V.N., Hatfield D.L. (2006). Thioredoxin Reductase 1 Deficiency Reverses Tumor Phenotype and Tumorigenicity of Lung Carcinoma Cells. J. Biol. Chem..

[96] Lubos E., Loscalzo J., Handy D.E. (2011). Glutathione Peroxidase-1 in Health and Disease: From Molecular Mechanisms to Therapeutic Opportunities. Antioxid. Redox Signal..

[97] Liu J., Hinkhouse M.M., Sun W., Weydert C.J., Ritchie J.M., Oberley L.W., Cullen J.J. (2004). Redox Regulation of Pancreatic Cancer Cell Growth: Role of Glutathione Peroxidase in the Suppression of the Malignant Phenotype. Hum. Gene Ther..

[98] Krehl S., Loewinger M., Florian S., Kipp A., Banning A., Wessjohann L.A., Brauer M.N., Iori R., Esworthy S., Chu F.-F. (2011). Glutathione peroxidase-2 and selenium decreased inflammation and tumors in a mouse model of inflammation-associated carcinogenesis whereas sulforaphane effects differed with selenium supply. Carcinogenesis.

[99] Banning A., Florian S., Deubel S., Thalmann S., Müller-Schmehl K., Jacobasch G., Brigelius-Flohé R. (2008). GPx2 Counteracts PGE2 Production by Dampening COX-2 and mPGES-1 Expression in Human Colon Cancer Cells. Antioxid. Redox Signal..

[100] Florian S., Wingler K., Schmehl K., Jacobasch G., Kreuzer O.J., Meyerhof W., Brigelius-Flohé R. (2001). Cellular and subcellular localization of gastrointestinal glutathione peroxidase in normal and malignant human intestinal tissue. Free Radic. Res..

[101] Yu Y.P., Yu G., Tseng G., Cieply K., Nelson J., DeFrances M., Zarnegar R., Michalopoulos G., Luo J.-H. (2007). Glutathione Peroxidase 3, Deleted or Methylated in Prostate Cancer, Suppresses Prostate Cancer Growth and Metastasis. Cancer Res..

[102] Yang W.S., SriRamaratnam R., Welsch M.E., Shimada K., Skouta R., Viswanathan V.S., Cheah J.H., Clemons P.A., Shamji A.F., Clish C. (2014). Regulation of Ferroptotic Cancer Cell Death by GPX4. Cell.

[103] Short S.P., Williams C.S., Tew K.D., Galli F. (2017). Chapter Two—Selenoproteins in Tumorigenesis and Cancer Progression. Advances in Cancer Re-search.

[104] Marciel M.P., Hoffmann P.R., Tew K.D., Galli F. (2017). Chapter Three–Selenoproteins and Metastasis. Advances in Cancer Research.

[105] Stockwell B.R., Jiang X., Gu W. (2020). Emerging Mechanisms and Disease Relevance of Ferroptosis. Trends Cell Biol..

[106] Gaschler M.M., Stockwell B.R. (2017). Lipid peroxidation in cell death. Biochem. Biophys. Res. Commun..

[107] Cao J.Y., Dixon S.J. (2016). Mechanisms of ferroptosis. Cell. Mol. Life Sci..

[108] Dodson M., Castro-Portuguez R., Zhang D.D. (2019). NRF2 plays a critical role in mitigating lipid peroxidation and ferroptosis. Redox Biol..

[109] Takahashi N., Cho P., Selfors L.M., Kuiken H.J., Kaul R., Fujiwara T., Harris I.S., Zhang T., Gygi S.P., Brugge J.S. (2020). 3D Culture Models with CRISPR Screens Reveal Hyperactive NRF2 as a Prerequisite for Spheroid Formation via Regulation of Proliferation and Ferroptosis. Mol. Cell.

[110] Ota H. (2013). Microtechnology-based three-dimensional spheroid formation. Front. Biosci..

[111] Ubellacker J.M., Tasdogan A., Ramesh V., Shen B., Mitchell E.C., Martin-Sandoval M.S., Gu Z., McCormick M.L., Durham A.B., Spitz D.R. (2020). Lymph protects metastasizing melanoma cells from ferroptosis. Nat. Cell Biol..

[112] Arnér E.S.J., Sies H. (2020). Chapter 31—Perspectives of TrxR1-based cancer therapies. Oxidative Stress.

[113] Zheng X., Chen Y., Bai M., Liu Y., Xu B., Sun R., Zeng H. (2019). The antimetastatic effect and underlying mechanisms of thioredoxin reductase inhibitor ethaselen. Free Radic. Biol. Med..

[114] Stafford W.C., Peng X., Olofsson M.H., Zhang X., Luci D.K., Lu L., Cheng Q., Trésaugues L., Dexheimer T.S., Coussens N.P. (2018). Irreversible inhibition of cytosolic thioredoxin reductase 1 as a mechanistic basis for anticancer therapy. Sci. Transl. Med..

[115] Anestål K., Arnér E. (2003). Rapid Induction of Cell Death by Selenium-compromised Thioredoxin Reductase 1 but Not by the Fully Active Enzyme Containing Selenocysteine. J. Biol. Chem..

[116] Anestål K., Prast-Nielsen S., Cenas N., Arnér E.S.J. (2008). Cell Death by SecTRAPs: Thioredoxin Reductase as a Prooxidant Killer of Cells. PLoS ONE.

[117] Ingold I., Berndt C., Schmitt S., Doll S., Poschmann G., Buday K., Roveri A., Peng X., Freitas F.P., Seibt T. (2018). Selenium Utilization by GPX4 Is Required to Prevent Hydroperoxide-Induced Ferroptosis. Cell.

[118] Niture S., Jaiswal A.K. (2012). Nrf2 Protein Up-regulates Antiapoptotic Protein Bcl-2 and Prevents Cellular Apoptosis. J. Biol. Chem..

[119] Latrèche L., Duhieu S., Touat-Hamici Z., Jean-Jean O., Chavatte L. (2012). The differential expression of glutathione peroxidase 1 and 4 depends on the nature of the SECIS element. RNA Biol..

[120] Papp L.V., Lu J., Bolderson E., Boucher D., Singh R., Holmgren A., Khanna K.K. (2010). SECIS-Binding Protein 2 Promotes Cell Survival by Protecting Against Oxidative Stress. Antioxid. Redox Signal..

[121] Gouge J., Satia K., Guthertz N., Widya M., Thompson A., Cousin P., Dergai O., Hernandez N., Vannini A. (2015). Redox Signaling by the RNA Polymerase III TFIIB-Related Factor Brf2. Cell.

[122] Faresse N.J., Canella D., Praz V., Michaud J., Romascano D., Hernandez N. (2012). Genomic Study of RNA Polymerase II and III SNAPc-Bound Promoters Reveals a Gene Transcribed by Both Enzymes and a Broad Use of Common Activators. PLoS Genet..

[123] Schramm L. (2002). Recruitment of RNA polymerase III to its target promoters. Genes Dev..

[124] Gouge J., Guthertz N., Kramm K., Dergai O., Abascal-Palacios G., Satia K., Cousin P., Hernandez N., Grohmann D., Vannini A. (2017). Molecular mechanisms of Bdp1 in TFIIIB assembly and RNA polymerase III transcription initiation. Nat. Commun..

[125] Lockwood W.W., Chari R., Coe B.P., Thu K.L., Garnis C., Malloff C.A., Campbell J., Williams A.C., Hwang D., Zhu C.-Q. (2010). Integrative Genomic Analyses Identify BRF2 as a Novel Lineage-Specific Oncogene in Lung Squamous Cell Carcinoma. PLoS Med..

[126] Bian Y., Li Q., Li Q., Pan R. (2020). Silencing of BRF2 inhibits the growth and metastasis of lung cancer cells. Mol. Med. Rep..

[127] Sanchez-Garcia F., Villagrasa P., Matsui J., Kotliar D., Castro V., Akavia U.-D., Chen B.-J., Saucedo-Cuevas L., Barrueco R.R., Llobet-Navas D. (2014). Integration of Genomic Data Enables Selective Discovery of Breast Cancer Drivers. Cell.

[128] Cabarcas-Petroski S., Meneses P.I., Schramm L. (2020). A meta-analysis of BRF2 as a prognostic biomarker in invasive breast carcinoma. BMC Cancer.

[129] Jameson R.R., Carlson B.A., Butz M., Esser K., Hatfield D.L., Diamond A.M. (2002). Selenium Influences the Turnover of Selenocysteine tRNA[Ser]Sec in Chinese Hamster Ovary Cells. J. Nutr..

[130] Gouge J., Vannini A. (2018). New tricks for an old dog: Brf2-dependent RNA Polymerase III transcription in oxidative stress and cancer. Transcription.

[131] Zucker S.N., Fink E.E., Bagati A., Mannava S., Bianchi-Smiraglia A., Bogner P.N., Wawrzyniak J.A., Foley C., Leonova K.I., Grimm M.J. (2014). Nrf2 Amplifies Oxidative Stress via Induction of Klf9. Mol. Cell.

[132] Yang L., Wang H., Yang X., Wu Q., An P., Jin X., Liu W., Huang X., Li Y., Yan S. (2020). Auranofin mitigates systemic iron overload and induces ferroptosis via distinct mechanisms. Signal Transduct. Target. Ther..

[133] Panieri E., Buha A., Telkoparan-Akillilar P., Cevik D., Kouretas D., Veskoukis A., Skaperda Z., Tsatsakis A., Wallace D., Suzen S. (2020). Potential Applications of NRF2 Modulators in Cancer Therapy. Antioxidants.

